# RAWS4all project: validation of a new silicone model for robotic TAPP inguinal hernia repair

**DOI:** 10.1007/s00464-023-10592-y

**Published:** 2023-12-18

**Authors:** Mário Rui Gonçalves, Salvador Morales-Conde, Sofia Gaspar Reis, Palmira Carlos Alves, José Novo de Matos, António Oliveira, Ricardo Marinho, Irene Cadime, Miguel Castelo-Branco Sousa

**Affiliations:** 1 Faculty of Health Sciences, University of Beira Interior, Av. Infante D. Henrique, 6200-506 Covilhã, Portugal; 2 Department of Surgery, University Hospital Virgen Macarena, University of Sevilla, Seville, Spain; 3 Centro Hospitalar Barreiro Montijo, Avenida Movimento das Forças Armadas, 2830-003 Barreiro, Portugal; 4 Curricular Studies and Educational Technology, Institute of Education, University of Minho, Campus de Gualtar, 4710-093 Braga, Portugal; 5 Centro Hospitalar Universitário Lisboa Central, Hospital de São José, Rua José Serrano, 1150-199 Lisbon, Portugal; 6 Centro Hospitalar de Trás-Os-Montes e Alto Douro, E.P.E, Av. da Noruega, 5000-508 Vila Real, Portugal; 7 Centro Hospitalar de Leiria, Hospital de Santo André, Rua das Olhalvas, 2410-197 Leiria, Portugal; 8 Research Centre on Child Studies, Institute of Education, University of Minho, Campus de Gualtar, 4710-057 Braga, Portugal; 9 Centro Academico Clinico das Beiras (Academic Clinical Center of Beiras), Faculty of Health Sciences, University of Beira Interior, Av. Infante D. Henrique, 6200-506 Covilhã, Portugal

**Keywords:** Surgical education, Simulation, TAPP, Assessment, Abdominal wall, Robotic surgery

## Abstract

**Background:**

Trans-abdominal pre-peritoneal (TAPP) hernia repair is a complex procedure that presents several challenges. Even though, due to the high prevalence of inguinal hernia, TAPP technique is increasing in frequency and robotic Abdominal Wall Surgery (rAWS) is emerging as a valuable tool in this regard. Although inguinal TAPP procedure principles have been published and simulation is needed, the availability of validated models remains scarce.

**Methods:**

A new low-cost model was developed to simulate inguinal rTAPP repair. For validity assessment, a new TAPP-specific fidelity questionnaire and assessment scale were developed to compare the performance of novices and experts in the simulated procedure. The models used were assessed at 60 min for execution and quality score.

**Results:**

Twenty-five residents and specialists from all over the country participated in this study. Execution, quality, and global performance was higher in the seniors group compared to juniors (8.91 vs 6.36, *p* = 0.02; 8.09 vs 5.14, *p* < .001; and 17 vs. 11,5, *p* < .001, respectively). Overall fidelity was assessed as being very high [4.41 (3.5–5.0), *α* = .918] as well as face [4.31 (3.0–5.0), *α* = .867] and content validity [4.44 (3.2–5.0), *α* = .803]. Participants strongly agreed that the model is adequate to be used with the DaVinci® Robot [4.52 (3.5–5.0), *α* = .758].

**Conclusion:**

This study shows face, content, and construct validity of the model for inguinal TAPP simulation, including for robotic surgery. Therefore, the model can be a valuable tool for learning, understanding, practicing, and mastering the TAPP technique prior to participating in the operating room.

**Graphical abstract:**

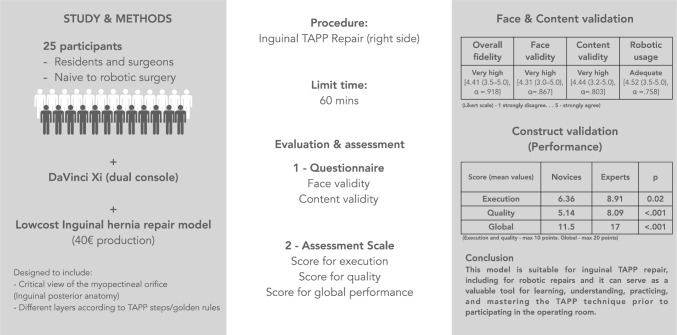

**Supplementary Information:**

The online version contains supplementary material available at 10.1007/s00464-023-10592-y.

Abdominal Wall Repair (AWR) procedures, particularly inguinal hernia repair [1],are among the most commonly performed surgeries worldwide [2, 3]. In the last decades, mostly in recent years, new techniques [4] and anatomical concepts [5] have emerged to enhance our understanding of AWR [6]. These showed a need for increased surgical volume and more and better training in this field. Regarding Minimally Invasive Surgery (MIS) for inguinal hernia, the two most commonly performed approaches are trans-abdominal pre-peritoneal (TAPP) followed by totally extra-peritoneal (TEP) repair with several benefits in comparison to open repair [2, 7, 8]. However, several factors, including the complexity of the procedures and limited knowledge of posterior (endoscopic) inguinal anatomy among most surgeons, specially residents, have limited their widespread adoption [5]. As soon as these challenges are resolved, the MIS approach for these hernias can probably be considered as the gold standard due to its advantages over the open approach and the huge prevalence of inguinal hernia [1]. Robotic surgery, particularly in the United States [9], is constantly growing and robotic Abdominal Wall Surgery (rAWS) has seen a rise in reported cases in the literature [10], demonstrating comparable results to other minimally invasive approaches in terms of postoperative complications [11, 12] and recurrence rates [13]. The first robotic hernia repair was described in 2007 [14] and since then, rAWS increased several folds [15]. Sheetz et al. [16] reported a remarkable increase in robotic inguinal hernia repair, from 0.7 to 28.8% (41.1-fold change) and robotic ventral hernia repair, from 0.5 to 22.4% (44.8-fold change) in just 6,5 years. Muysoms [9] considered robotic groin hernia repair, specially robotic TAPP (rTAPP), as an important initial procedure in the training of AWR surgeons, allowing for learning and training dissection, suturing, and mesh handling. As the robot usage continues to grow, the demand for training and certification also increases, especially with the introduction of new systems to the market. As in other areas, simulation plays an important role [17], however, in the field of Robotic Surgery, simulation-based curricula are even more crucial due to limited access to the robotic platforms, fewer training opportunities, high costs and logistical challenges associated with wet-lab sessions. Simulation models are often expensive and it is important to develop cost-effective simulators [17], assess them for face, content, and construct validity, as well as their educational impact. In the field of AWR, twenty-one laparoscopic hernia repair models were described, [18] but only 4 models for laparoscopic inguinal hernia TAPP repair. Overall, these authors concluded that only a few models demonstrated total validity and educational impact. Some of these models appeared complex to construct and showed a limited appeal to experienced surgeons. Most of them lacked construct validity; didn’t show similar characteristics to the real anatomy/procedure; and only one showed the transfer of skills from simulation to the operating room. These issues are recurrent problems faced by simulation models and attempting to simulate an entire procedure is often not possible. Therefore, it is advisable to break down the procedure to some of its core steps [19]. Regarding TAPP inguinal repair, in 2017, Daes and Felix [6] described the “Critical View of the myopectioneal orifice” (MPO) and two years later, Furtado et al. [5] systematized the procedure based on a new anatomical concept. They idealized an inverted Y, based on the relations of the groin anatomical landmarks and described 5 triangles inside the MPO, with 3 dissection zones. Subsequently, in 2020, Claus et al. [20] described and systematized the rules to master and perform TAPP hernia repair in a safe way. These groups advocate that, in general, the main steps of the procedure were: 1) peritoneal incision; 2) creation of the peritoneal flap with dissection of the pre-peritoneal plane; 3) mesh placement with or without fixation; 4) closure of the peritoneum using absorbable or barbed sutures. They also highlighted that the dissection of the central zone (zone 3) was the most demanding step, requiring careful dissection to avoid errors and injury to critical structures such as the iliac vessels or cord elements. Having in mind the need and challenges of surgical training, specially in robotic surgery, the difficulty of posterior anatomy of the groin and the need of standardization for achieving desired safety and outcomes, we have developed a low-cost silicone model that enables the simulation of the critical view of the MPO and allows adherence to the golden rules of safe TAPP repair.

## Materials and methods

### Participants

This study was conducted among Portuguese surgical residents and specialists in order to assess a new MIS TAPP inguinal hernia repair simulation model. Twenty-five participants without experience in Robotic Surgery or Inguinal TAPP repair completed the full training program and were assessed during and at the end of the session.

### Study design

The present study is a prospective observational study based on performance assessment and opinions/perspectives about a new simulation model for robotic Trans-Abdominal Pre-Peritoneal (rTAPP) Inguinal Hernia repair. The study was carried out during an “Introduction to Robotic Surgery Course,” held at CUBI Surgical Simulation Center, University of Beira Interior, in April 2023. Course registration was free on a first-come, first-served basis, without active participant selection by the research team.

Firstly, participants were required to complete an online questionnaire regarding their demographic information and self-assessment of laparoscopic skills through the website www.lap-school.com. All participants consented to participate in the course and in the study. They were then instructed on the steps of the procedure, including the defined goals and criteria for minor/major errors. Afterward, participants simulated a rTAPP inguinal hernia repair using a new silicone model on the DaVinci Xi® system. After the course, they were asked to voluntarily and anonymously answer to a fidelity questionnaire.

### Statistics

Execution and Errors domains and the Full Procedure were scored following the assessment scale specific for the model. Questionnaire results on fidelity were collected. All statistical analyses were performed with the software IBM® SPSS Statistics 28 for MacOS®. After computing descriptive statistics, we examined the differences in the points (score) obtained by juniors and seniors in each domain and the full procedure running independent samples t-tests, after checking the normality of the distribution and homogeneity of variances. For fidelity we calculated means and standard deviation, and Cronbach α for the total questionnaire (14 questions), face-validity related questions (Q3-4 and Q13-14), content-validity related questions (Q5-8 and Q12) and model utility for robotic training in the DaVinci Xi® system (Q1-2 and Q9-10).

### Model design

**Fig. 1:**
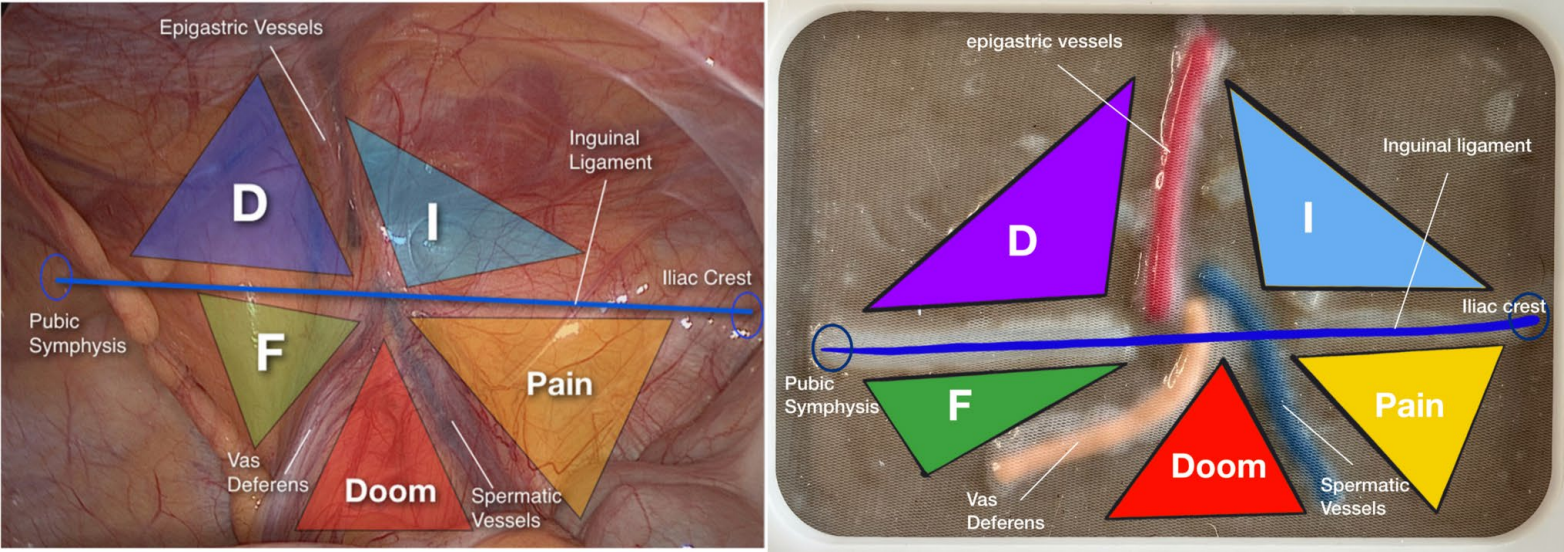
Left—Original figure from Furtado et al. (reproduced with written authorization of the Author) with the representation of the 5 triangles, the “inverted Y” elements and the inguinal ligament. Right—The 5 triangles represented in our model with indication of the same elements as the original report

Our model represents the posterior view of the inguinal anatomy, on the right side. Based on the “Inverted Y + 5 triangles concept” proposed by Furtado et al. [5] it includes 4 crucial anatomical landmarks and 2 structures representing the same concept as described Fig. 1.

### Model production

Our model is totally handmade, using different silicone types and mixtures, colored differently, according to the anatomical elements represented on it. For the creation of the model, we firstly designed and produced molds of the different elements.

These elements compose the 3 layers of the model:

- Layer 1—*“Structure 1”*—Abdominal Wall/Transversalis fascia—made of silicone “A” + pigment (pink/yellow/brown mixture)
- Layer 2—*“4 Anatomical landmarks”*—epigastric vessels, vas deferens, spermatic vessels, inguinal ligament—made of silicone “B” + pigments (red, skin, blue, and white)
- Layer 3—*“Structure 2”*—Peritoneum—textile mesh and silicone “A” + “B” + pigment (white)

**Fig. 2:**
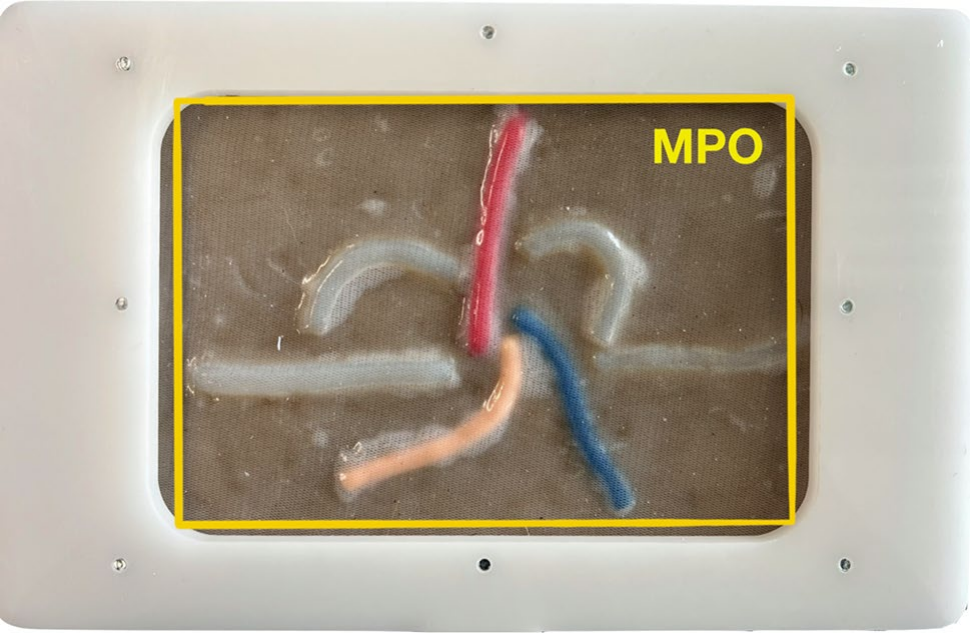
Final model + structure creating a window for MPO dissection

The elements of Layer 2 are placed over Layer 1 following the inverted Y concept and accordingly to the posterior anatomy of the MPO and thus developing the 5 triangles described. Layer 3 is finally placed over the other two layers and after cure, the silicone model is secured on an acrylic, laser-cut structure designed to support the silicone model creating a 17 × 11.5 cms “window,” representing the MPO, thus indicating the area that should be dissected for an adequate mesh placement Fig. 2.

The production includes three stages and total time for each model production is approximately 4 h, including the time to cure the silicones. The model is single-use and the estimated cost is 40 €/model.

### Training program

**Fig. 3:**
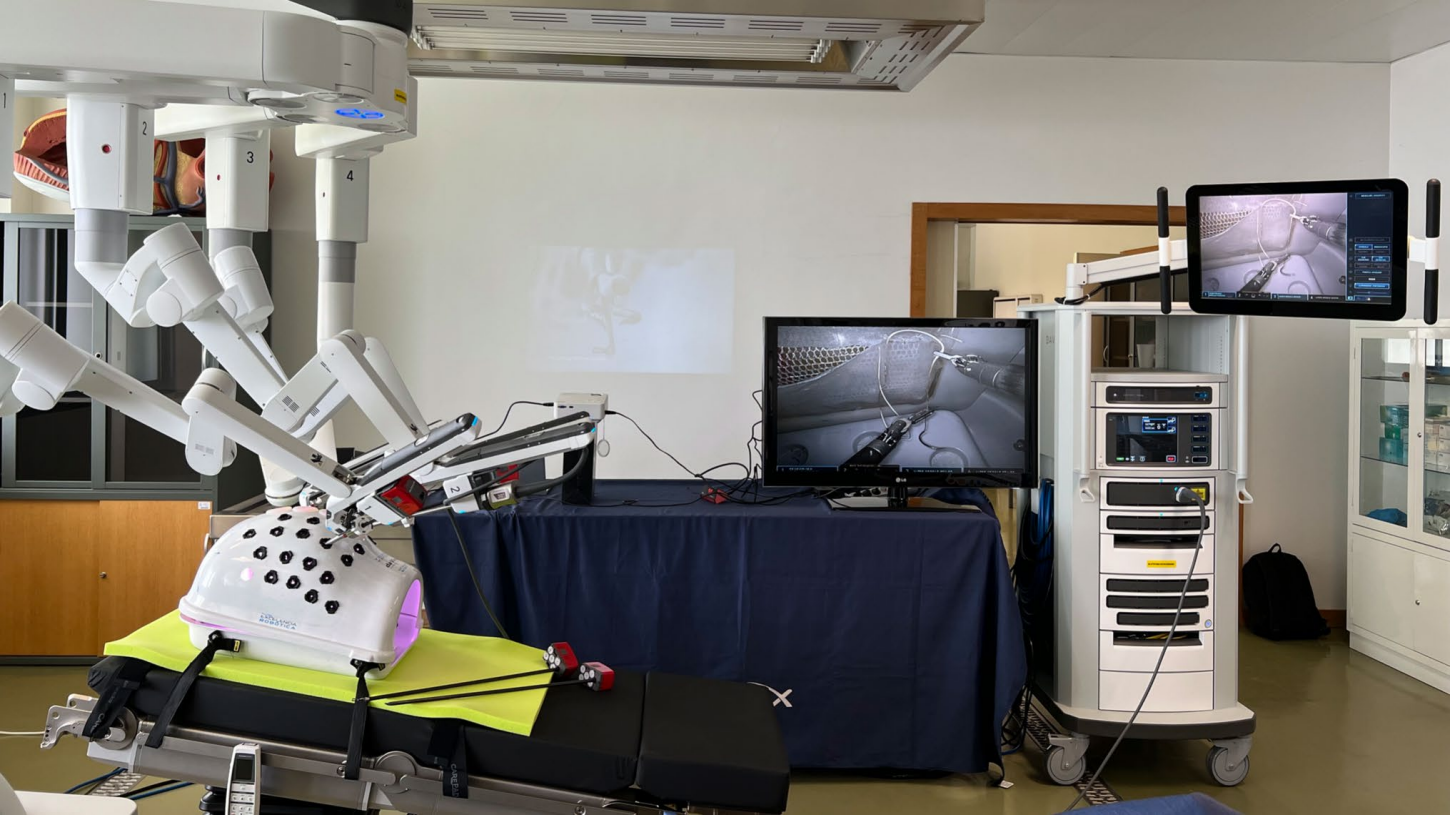
DaVinci Xi® docked during the simulation (in the image the participant was beginning the closure of the peritoneum—step 4)

**Fig. 4:**
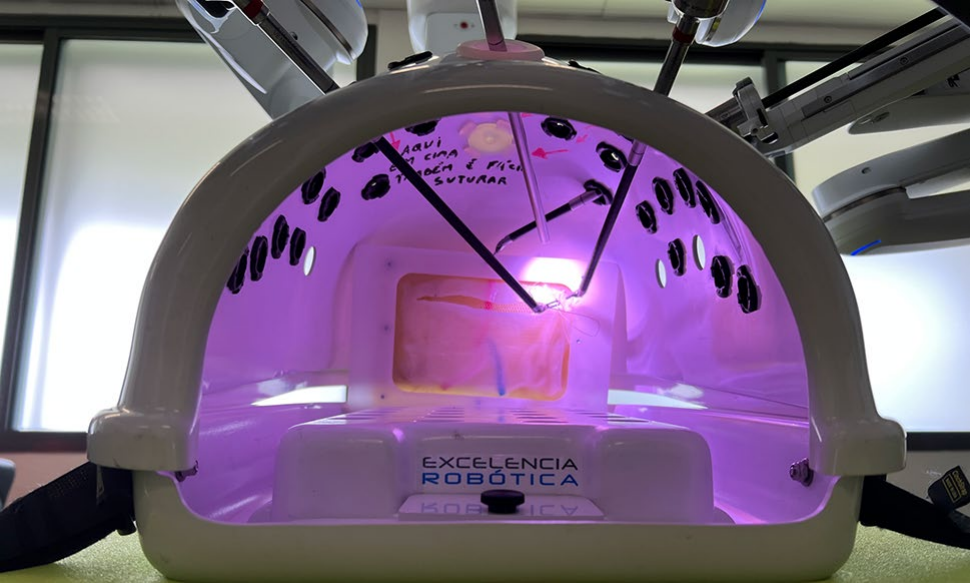
Visualization of the model inside the DaVinci Xi® endotrainer/box (as in Fig. 4, the participant was beginning the closure of the peritoneum—step 4)

For this simulation, all the participants played the role of console surgeons on the DaVinci Xi® system. At the beginning of each session, a model + structure was placed on the DaVinci® endotrainer/box and the robot is docked—Fig. 3 at an angle mimicking the posterior inguinal region for TAPP repair Fig. 4.

**Fig. 5:**
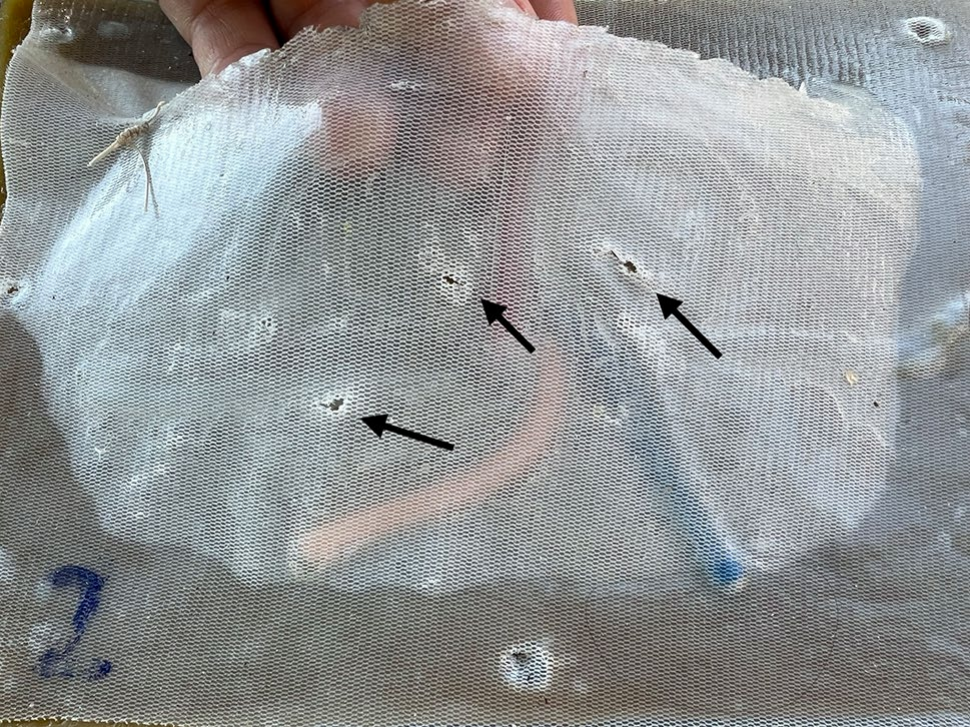
Peritoneal tears-minor error

**Fig. 6:**
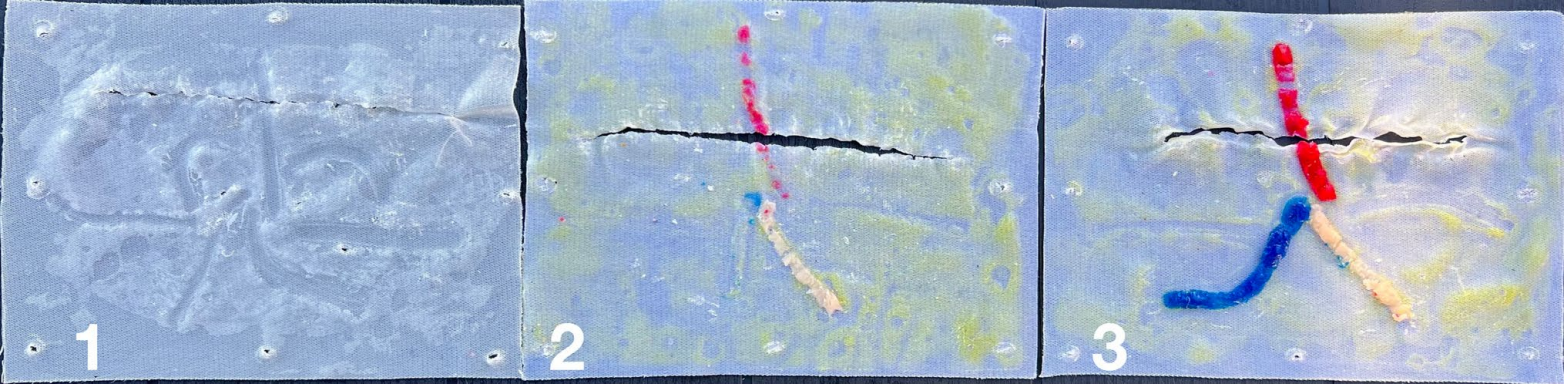
1) Benchmark; 2) minor injuries to cord elements (minor errors); 3) major injuries to cord elements and artery (major errors)

**Fig. 7:**
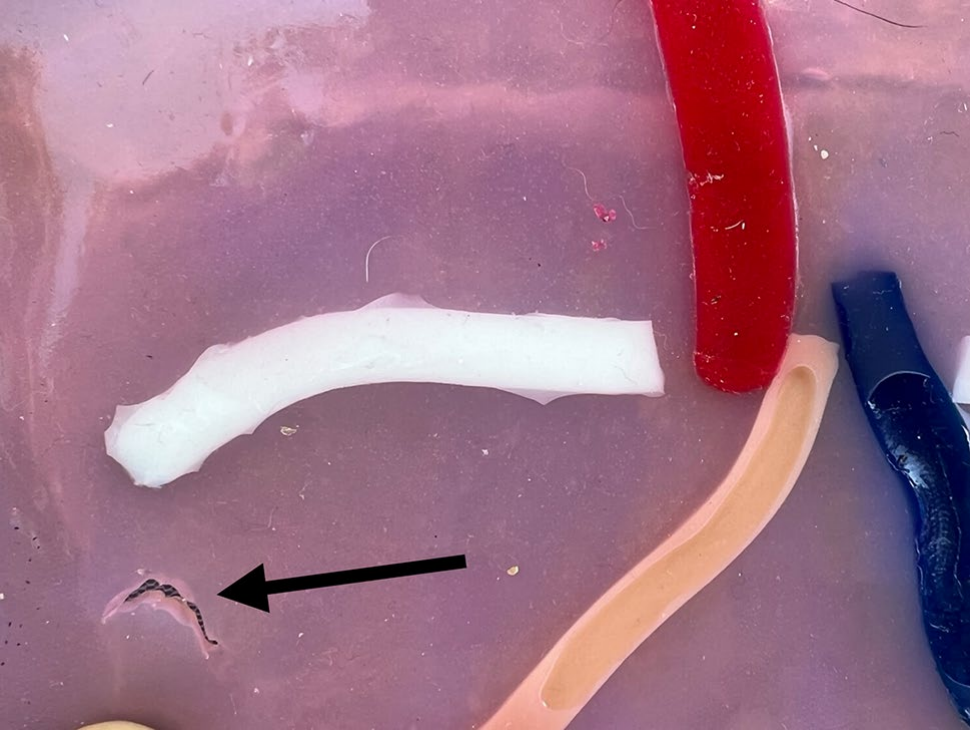
Abdominal wall injury (major error)

We adopted the same training program as previously described by our team [21]. Our validated introduction to Robotic surgery training program consists of two parts: Session #1—Virtual exercises (1 h) on the DaVinci® simulator console; and Session #2—Anatomical model simulation (1 h) with the DaVinci Xi® robotic system. After Session #1, the mentor/observer pointed directly on the system monitor the anatomical landmarks; explained the steps and the good principles of the rTAPP inguinal hernia repair procedure; enumerated the objectives of the simulation; and explained what was considered as minor and major errors that should be avoided for the execution of a safe and high quality procedure Figs. 5, 6 and 7; For this simulation, 3 arms of the robot were used with 4 instruments, arm 1—Grasper; arm 2—camera; arm 3—Scissors/Needle-holder—Table 1.

**Table 1:** Description of the model, training objectives, exercise steps, minor and major errors and classification

Inguinal hernia rTAPP repair model		
Description	The trainee is presented with a silicone model of the posterior view of the inguinal region, including the peritoneum, inferior epigastric artery, vas deferens, spermatic vessels, inguinal ligament and abdominal wall	
Training objectives	Surgical skills: cutting, grasping, traction, dissection, mesh placement and extension, suture	
	Robotic skills: control of 2 arms and camera	
Execution (Exercise steps)	The trainee is required to perform a rTAPP inguinal hernia repair in less than 60 min	(Points are given if Goal is achieved) Total of 10 points
	The procedure should start with a linear section (Goal 1) of the peritoneum at ∼ 3–4 cms above the represented inguinal ligament (Goal 2) within the limits of the model (Goal 3*)*	4 points
	The pre-peritoneal space should be dissected as near as possible to the limits of the acrylic structure to create the peritoneal flap (Goal 4)	1 point
	A mesh should be placed and fully expanded into the dissected space (Goal 5)	2 point
	The peritoneum is then totally closed with a non-barbed suture (Goal 6) and finished with a double knot (Goal 7)	3 points
Quality (Minor errors)	Trainees should avoid:	(Points are given if absense of errors)—Total of 6 points
	Producing tears on the represented peritoneum (Minor error 1)	1 point
	Leaving the mesh folded (Minor error 2)	2 points
	Irregular size of stitches (Minor error 3)	1 point
	Irregular space between stitches (Minor error 4*)*	1 point
	Big “bites” (stitches) (Minor error 5*)*	1 point
Quality (Major errors)	Trainees should avoid:	(Points are given if absense of errors)—Total of 4 points
	Injuries to the represented cord elements and artery (Major error 1)	2 points
	Injuries to the Abdominal wall (transversalis fascia) (Major error 2)	2 points

### Procedure steps

As in real life, the simulation starts with the incision of the peritoneum using the scissors (step 1). After this, pre-peritoneal space is dissected using the grasper and the scissors (step 2). Special attention should be taken for adequate and sufficient traction of the “peritoneal tissue” with the left arm to facilitate flap creation and individualization of the cord elements. Dissection and cutting with the right arm should be performed carefully and precisely to minimize errors. Once the space is totally dissected, a mesh is placed, ensuring it lies flat on the abdominal wall and fully covers the MPO (step 3). In this study we used a 15 × 10 cms Progrip® mesh. Finally, the peritoneum is closed with a running suture (step 4). At 60 min of simulation, the observer analyzed each model, photographed it and evaluated them accordingly to the assessment scale Fig. 8 Video(sup. file).

**Fig. 8:**
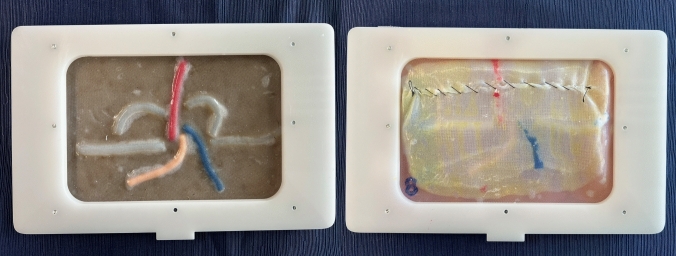
Pre- and post-simulation model

### Fidelity questionnaire

Based on Rehmann et al. [22] and LeBlanc et al. [23]’s work, we developed a new questionnaire, specially designed for this model and training program. We selected 14 questions/statements for evaluation of *environment*, *equipment* and *psychological* domains and thus evaluate face and content validity of the new TAPP model, based on a five-point Likert scale (1-strongly disagree; 2-disagree; 3-neither agree or disagree; 4-agree; 5-strongly agree) Table 2.

**Table 2:** Fidelity questionnaire

Fidelity-environment	
Q.1	The use of the inguinal TAPP model in the robotic system wasn’t problematic
Q.2	This TAPP model is suitable for using with the DaVinci Robotic System
Q.3	Visual representation of the inguinal anatomy was realistic enough for the procedure
Q.4	Visual representation of the measures of the Myopectineal Orifice of Fruchaut is important for the performance of this procedure (17 × 11.5 cms area)
Fidelity-equipment	
Q.5	This model allows the simulation of PERITONEAL INCISION (not talking about difficulty)
Q.6	This model allows the simulation of PRE PERITONEAL SPACE DISSECTION (not talking about difficulty)
Q.7	This model allows the simulation of PLACEMENT OF THE MESH (not talking about difficulty)
Q.8	This model allows the simulation of PERITONEAL CLOSURE (not talking about difficulty)
Fidelity-psychological	
Q.9	I felt comfortable performing the simulation
Q.10	I felt like all my senses were engaged during the simulation
Q.11	The events and environment around me made me feel like I was actually performing surgery
Q.12	In general, while performing this simulation it felt like I was actually doing this surgical procedure
Q.13	This exercise made me feel like I was actually performing a real inguinal hernia repair
Q.14	The visual aspects of the model made me feel as if I was actually performing a TAPP repair

## Results

Twenty-five participants (*n* = 25), 19 females (*n* = 19) and 6 males (*n* = 6) completed the full training program. As all the participants were inexperienced in TAPP repair and Robotic Surgery, the sample was divided into 2 groups (juniors and seniors) based on a pre-course self-assessment questionnaire on experience in general surgery and laparoscopy. Characteristics of the participants are presented on Table 3.

**Table 3:** Characteristics of the participants

	Total	Gender	
		Female	Male
1Y resident	5 (20%)	2 (8%)	3 (12%)
2Y resident	3 (12%)	2 (8%)	1 (4%)
3Y resident	5 (20%)	5 (20%)	0 (0%)
4Y resident	1 (4%)	1 (4%)	0 (0%)
5Y resident	4 (16%)	3 (12%)	1 (4%)
6Y resident	3 (12%)	3 (12%)	0 (0%)
Surgeon/specialist	4 (16%)	3 (12%)	1 (4%)
Total	25 (100%)	19 (76%)	6 (24%)
Surgical Experience	“*I have no/little laparoscopic experience****”***(juniors)	“*I have some/high laparoscopic experience****”***(seniors)	
	14 (56%)	11 (44%)	
Inguinal TAPP repair experience	No	Yes	
As assistant or observer	22 (88%)	3 (12%)	
As surgeon	25 (100%)	0 (0%)	
Robotic Surgery experience	No	Yes	
As assistant or observer	25 (100%)	0 (0%)	
As surgeon	25 (100%)	0 (0%)	

## Fidelity questionnaire results

Twenty-two of the 25 (88%) attendants answered the anonymous online survey after the course. The overall fidelity (on a 1 to 5 Likert scale) of the questionnaire was 4.41 (3.5–5.0), *α* = 0.918. Regarding face and content validity questions/statements the results were 4.31 (3.0–5.0), *α* = 0.867 and 4.44 (3.2–5.0), *α* = 0.803, respectively. Participants strongly agreed that the model is well adapted to be used with the DaVinci Xi® Robot, 4.52 (3.5–5.0), *α* = 0.758—Table 4.

**Table 4:** Fidelity questionnaire results

Fidelity results						
	Questions	Number of items	Mean (SD)	Minimum	Maximum	Cronbach’s alpha
Face validity	3, 4, 13, 14	4	4.31 (0.58)	3.00	5.00	0.867
Content validity	5, 6, 7, 8, 12	5	4.44 (0.53)	3.20	5.00	0.803
Robot adequacy	1, 2, 9, 10	4	4.52 (0.46)	3.50	5.00	0.758
Total	1 to 14	14	4.41 (0.47)	3.50	5.00	0.918

## Procedure results

**Fig. 9:**
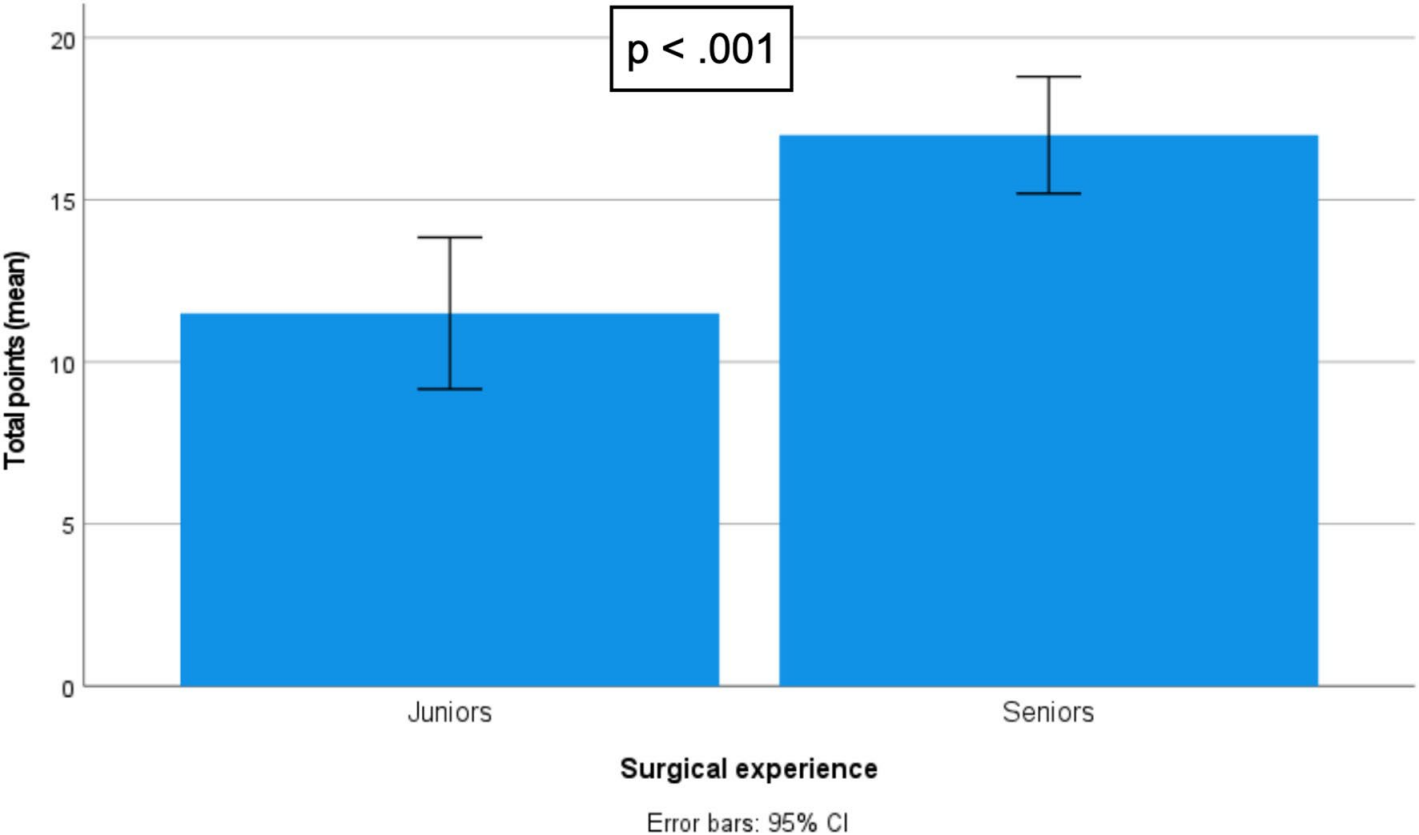
Score differences between juniors and experts: “total procedure.”

**Fig. 10:**
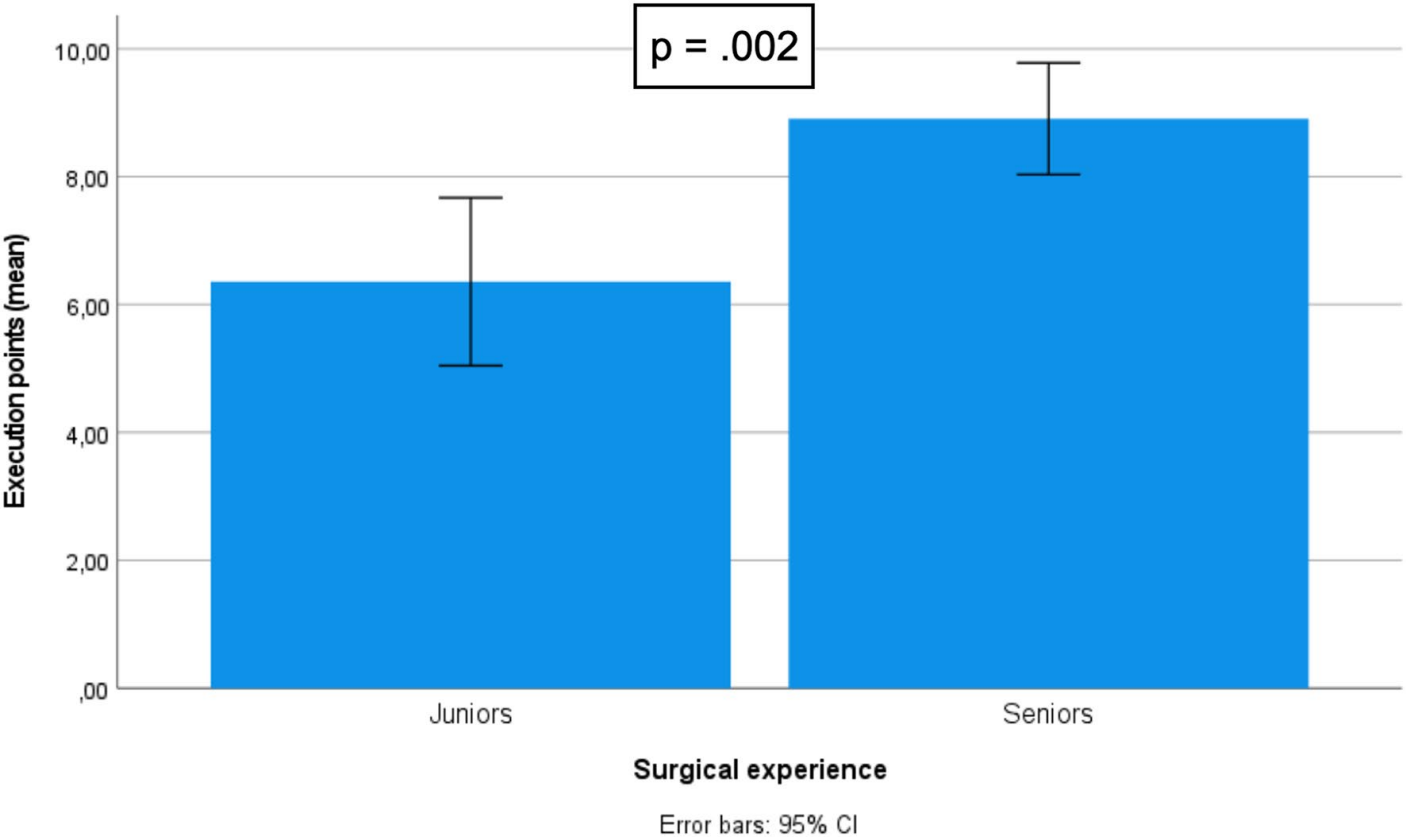
Score differences between juniors and experts: “execution = steps”

**Fig. 11:**
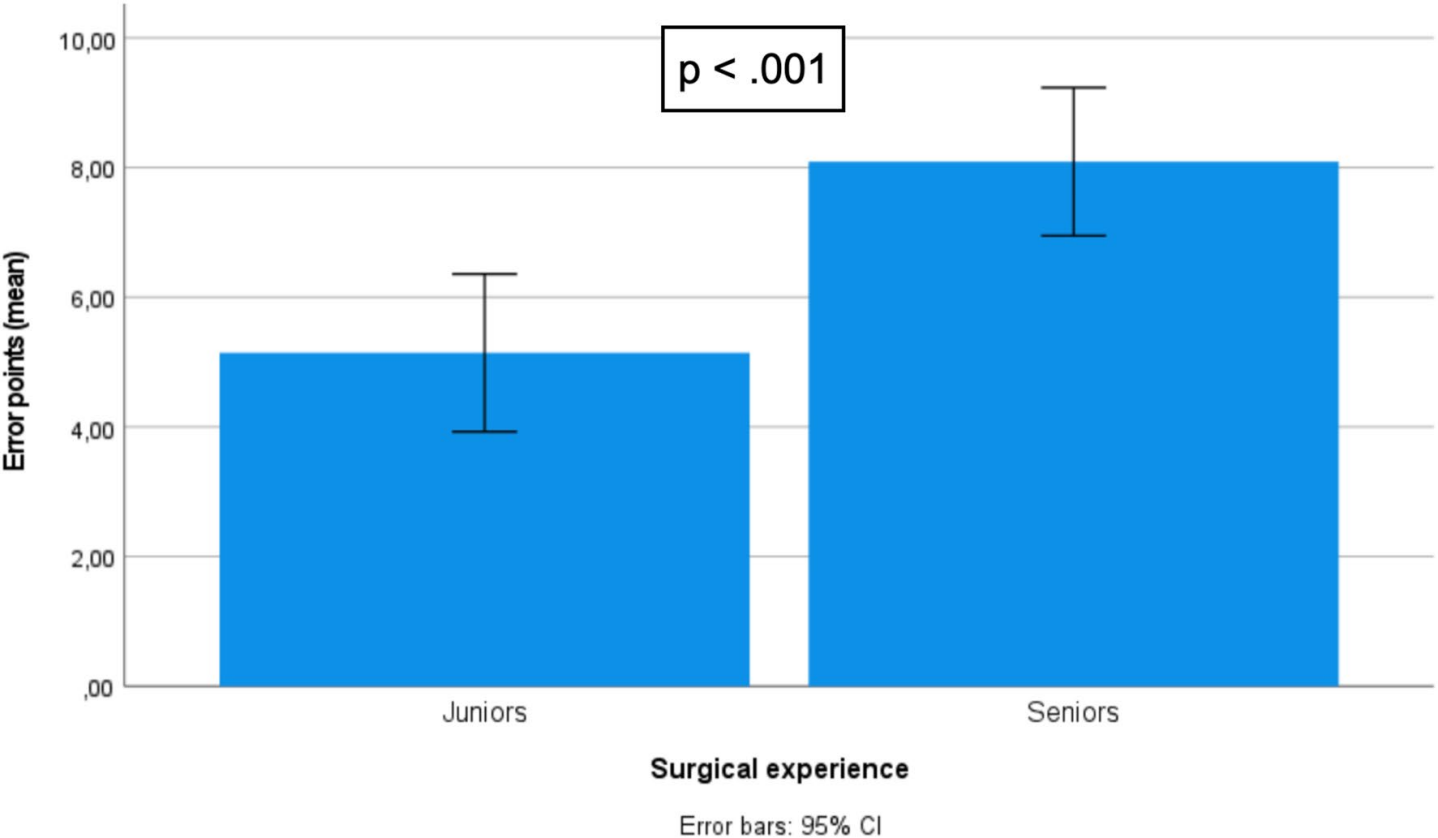
Score differences between juniors and experts: “quality = errors.”

The assessment was made to the model, at the end of the limit time (60 min). Higher scores in classification indicate better performance. Global performance was higher in the seniors compared to juniors (17 vs. 11,5 points out of 20, *p* < 0.001). Seniors had better performance during the execution (8.91 vs 6.36 points out of 10, *p* = 0.02) of the procedure and had less errors (8.09 vs 5.14 points out of 10, *p* < 0.001). The comparison results among groups are depicted in Table 5 and Figs. 9, 10 and 11.

**Table 5:** Procedure results

		Máx score	Mean (Std. Dev.)	*P*-value^a^
Score execution (Exercise steps)	Juniors	10	6.36 (2.27)	0.002
	Seniors	10	8.91 (1.30)	
Score quality (Minor + Major errors)	Juniors	10	5.14 (2.11)	< 0.001
	Seniors	10	8.09 (1.70)	
Score total procedure	Juniors	20	11.50 (4.05)	< 0.001
	Seniors	20	17.00 (2.68)

## Discussion

Recently, abdominal wall surgery has been recognized as a subspecialty by the Unión Européenne des Médecins Spécialistes (UEMS), in collaboration with the European Hernia Society (EHS) [15] mostly because of a greater awareness of hernia clinical and community implications, complexity of the abdominal wall anatomy, development and description of new procedures and materials for hernia repair. Nowadays, a minimum number of procedures to achieve proficiency in TAPP repair is not well established yet [2, 3, 10] although some groups defend around 50–100 procedures as the minimum number for TAPP learning curve [24]. Undoubtedly there is a need for the development of new training opportunities and simulation models in order to establish a more realistic number and, if possible, to reduce the learning curve for TAPP and the UEMS/EHS Fellowship of the European Board of Surgery-Abdominal Wall Surgery (FEBS-AWS) accreditation may play an important role. On the other side, there are real challenges for training and surgery proficiency such as working legislation, legal and ethical concerns, and lack of proctors and simulation opportunities [17]. The three most important learning methods for hernia repair are participation in the OR and video-demonstrations followed by hands-on hernia courses [25]. That highlights the importance of hands-on simulation and the need to be widely accessible to residents and surgeons meaning that models should be low-cost and with low logistics; valuable from an educational point-of-view; and suitable for procedure and skills assessment. We developed a low-cost dry-lab endotrainer and started to develop specific low-cost models for training, such as this inguinal TAPP repair model, ventral IPOM and laparoscopic cholecistectomy procedures. We are starting to validate some of the models for our national training program for general surgery residents. As well as with laparoscopy, we are developing some curricula for robotic surgery. Competence in rAWS will depend on the surgeon’s overall experience, familiarity with the robotic platform, and, particularly, their experience in AWR. The main objective of a training pathway is to allow a safe introduction to clinical practice and to promote patient safety, ensuring acceptable outcomes from the surgeon’s very first case. This is specially true in the context of robotic surgery [26]. At the moment, the principal rAWS training pathway is controlled by Intuitive Surgical, Inc® and consists of 5 phases (TR100 and TR200-preclinical, TR300, TR400 and TR500—clinical) including online, video-based, hands-on and live surgery sessions with a proctor [27]. However, this pathway was designed for generic robotic surgery and it is not rAWS-specific so scientific societies such as the EHS should play the most important role in the design and implementation of these pathways [15]. In 2023, the Robotic4all Project [21] showed that a 2-h, non-procedure specific program can be useful for the initial phase of the robotic training pathway. Twenty-seven, robotic-naive, General Surgery residents and specialists performed suture 2.6 fold faster and with more precision in the robot training box than in the laparoscopic training box**.** Other programs, developed for other robotic systems, including virtual simulators, showed that simulation-based programs will continue to be key to surgical education [28], specially for learning and improve performance in Robotic Surgery in a patient safe environment [29]. Although our models have a low cost production, they are designed with a special focus on surgical concepts, procedure steps, and anatomical landmarks to make the simulation as accurate as possible and to allow skills assessment. Our TAPP model is validated for the First Trainer® but we wanted to know if it would be useful with the DaVinci® robot as robotic surgery is becoming more and more available. In this study we were able to show that not only the trainee can simulate the full procedure but he/she can be assessed on execution and quality of the simulation. The model was designed having in mind three of the most important papers about inguinal anatomy and MIS inguinal hernia repair (TAPP and TEP) [5, 6, 20]. The dissection of the pre-peritoneal space is the most difficult step of the TAPP procedure, because of the individualization of the cord elements, mandatory to create an adequate space for the mesh to be placed safely and adequately. A very positive aspect of our model is that the peritoneum layer (layer 3) is stick to the sub-layers (layer 2-cord elements and layer 1-abdominal wall) so it increases fidelity as trainees have to separate the peritoneum and dissect it from the structures, simulating the difficulty of the real dissection. This and the presence of the posterior view of the inguinal region, representing the three zones of dissection (specially the “Doom triangle” in “Zone 3” and the “Pain triangle” in “Zone 1”) allows for a better understanding of the procedure on a systematized way, knowing where the errors and difficulties may happen. Dissection in “Zone 2” and “Zone 1” is easier to perform but it must be paid attention to the presence of some important structures such as: corona mortis; direct and femoral hernia; and inguinal nerves (“Pain triangle”), respectively. Other positive aspect of our model is the presence of key anatomical landmarks, such as the epigastric artery, the vas deferens, the spermatic vessels and inguinal ligament allowing for the representation of the other 3 triangles (direct, indirect, femoral) which allows for a better understanding on the location of the 3 types of hernias in order to know how to look for their existence. In fact, the anatomical representation of the 5 triangles and the inverted Y allowed us to create a specific assessment scale for the procedure, with 2 domains: execution (adequate conclusion of steps, scored 0–10 points) and quality (absence of errors, scored 0–10 points) that could be totally and objectively assessed by the trainer and understood by the trainees. These two domains, summed up, result on the “procedure score” to assess the trainee globally (execution + quality, scored 0–20 points) and this is one of the differences of our assessment scale from other actual scales, like Global Operative Assessment of Laparoscopic Skills (GOALS) [30] or its adapted scale for groin hernia (GOALS-GH) [31]. Our course registration was free and on a first-come, first-served basis so we didn’t know who were the participants and their global, TAPP and robotic experience. Because of this, we weren’t able to send them any info or educational material before the course. Despite this, before the beginning of the simulation, the mentor reviewed the procedure steps; pointed out the anatomical landmarks and structures; explained the area that should be dissected (representing the MPO); and pointed out the errors that could happen and should be avoided. We believe that if this explanation was given some days before the procedure, supported with videos and other educational material would help trainees better understanding of TAPP procedure and scores could be even higher. For the following editions of the course we will have that into account. This opens the pathway to other studies regarding surgical education and assessment of our general surgery training program. Another limitation of the study is Robots availability for training, which is very low and is one of the main challenges that robotic surgery training faces, as we faced during our study. Although we think 25 participants is an adequate number, specially for a robotic surgery study, and is in line with similar studies, we only had one robot for the course so we didn’t have enough time to have more participants or to have more than 1 simulation per participant. Even though, the results showed that fidelity was very high in all aspects and it was possible to differentiate experts from novices in every domain of the simulation indicating construct validation of the model. It demonstrates that our model can be used for learning and practicing of residents and surgeons disregarding their previous experience and it can also be used as a tool for skills improvement assessment and skills maintenance. From costs point-of-view, in fact, we were able to offer twenty-five residents and surgeons the opportunity to learn and practice robotic inguinal hernia TAPP repair simulation for just a small investment of less than 1200 €, approximately.

## Conclusion

Specific training and assessment are key to better surgical competence, for skills transfer to the OR and, lastly to, better surgical outcomes. This study allowed us to validate our rTAPP model as a useful tool for TAPP learning, practicing, and assessment. It may play an important role for novice residents to learn TAPP hernia repair and for shortening the learning curve of senior residents and general surgeons. Additionally, it seams a valuable tool for trainees and trainers to assess and follow their improvement and it may be adopted by surgical societies or integrated in robotic surgery programs for hernia education and certification. These findings open up new lines of development for models and educational programs as well as the design of new tools for procedure-specific skills assessment.

### Supplementary Information

Below is the link to the electronic supplementary material.

Supplementary material 1 (MOV 14,811 kb)
